# Evaluating caregivers’ service quality perceptions: impact-range performance and asymmetry analyses

**DOI:** 10.1186/s12913-022-07594-2

**Published:** 2022-02-12

**Authors:** Wen-Fu Wang, Chun-Min Chen, Kai-Ming Jhang, Yung-Yu Su

**Affiliations:** 1 Department of Neurology, Changhua Christian Hospital, Changhua, Taiwan; 2 Department of Recreation and Holistic Wellness, Ming Dao University, Changhua, Taiwan; 3 Big Data Center, Changhua Christian Hospital, Changhua, Taiwan; 4 Department of Long-Term Care, National Quemoy University, No. 1, University Rd., Jinning Township, Kinmen County 892, Taiwan R.O.C, Kinmen, Taiwan

**Keywords:** Attribute-performance, Impact asymmetry, Customer satisfaction, Patients with dementia, Family caregiver

## Abstract

**Background:**

This study aimed to analyze family caregivers’ (FCs) dementia care service perceptions to identify the various attributes impacting FCs satisfaction and dissatisfaction.

**Methods:**

This is a cross-sectional survey study using convenience sampling methods. A self-completion questionnaire was developed from the Service Quality scale and distributed using a convenience sampling method to family caregivers in community-based dementia care centers to determine their perceptions of service quality in dementia care. Both exploratory factor analysis and reliability analysis were conducted to confirm the validity and factor structure of the scale. This study employed Impact Range Performance Analysis (IRPA) and Impact Asymmetry Analysis (IAA) to analyze the data obtained from FCs across five attribute dimensions (Tangibles, Reliability, Responsiveness, Assurance, and Empathy). Priorities for service improvement were derived using a three-step analytical framework.

**Results:**

This study reported that the overall perceived performance of service provided is high. The results indicated that practitioners should focus on attributes such as demand coordination, appropriate services, timely service, barrier-free environment, care-giving process, fire and safety compliance, professional knowledge, and reliable services, which have a higher range of impact on customer service and low impact-asymmetry and attribute performance scores.

**Conclusion:**

This study used expectation and perceived performance to suggest that the priorities for improvement and resource allocation in dementia care centers vary across different attributes. Thus, attentiveness toward satisfying user demand could improve patient care and caregiver satisfaction. The dimensions and attributes identified by our study can serve as basic data for future research on the long-term care system.

## Background

Long-term care (LTC) services are the future policy of global health care development in response to the aging population [1–3]. The World Health Organization declared Taiwan an "aged society" in 2018, and predicted that in 2026, it will become a "super-aged society." Additionally, the number of people with dementia in Taiwan exceeded 280,000 by the end of 2019 [4]. Consequently, the Ministry of Health and Welfare accelerated the promotion of relevant policies in response to the rapid growth of this population [3]. "LTC 2.0" is the newer version of the project, which was launched in early 2017. The LTC 2.0 policy specifically targets people with dementia, aged over 50 years, as service consumers, and specifies dementia care as the top priority. The drastically changing utilization rate of various dementia care services, necessitates the establishment of an LTC service database. Furthermore, it is essential to survey the service quality to obtain the relevant statistics and understand the impact of these services on the family and the society.

Family caregivers have an important role in providing support and care for relatives with dementia [5]. Family caregivers (FCs) may refer to unpaid family member, friends, or neighbors who provide assistance for at-home care delivery and assist in the activities of daily living [6]. In addition to assisting with important care responsibilities, they participate in advocating and arranging various healthcare services [7]. Therefore, family caregivers’ perspectives are of high importance as this help to better describe users' need and identify potential ways to improve dementia care service. Existing literature on LTC services involves many studies exploring users' perceptions [8, 9]. However, very little consideration has been given to the perceptions of family caregivers’ on community-based service in supporting dementia care [5, 10]. Assessing the experience of service use among family caregivers will help to delivering better quality dementia care to the loved one.

Recent studies have assessed users’ experiential quality by employing impact-range performance analysis (IRPA) and impact asymmetry analysis (IAA) [11]. Furthermore, these methods have been employed as suitable alternatives in some studies in the fields of hospitality, tourism, and consumer behavior [12, 13]. However, due to the focus on service industries, for researching service quality gaps, the literature on experiential quality is limited among primary caregivers [14], and it is mainly directed toward patients receiving primary care in hospitals or homes [15, 16]. Moreover, existing research has not investigated the relative importance of different service attributes—underperforming attributes acceptable to customers, attributes requiring higher performance, and those that must be prioritized for interventions—in facilitating user caregiver satisfaction. Therefore, it is essential to assess FCs underlying needs to bridge the existing research gap and offer potential managerial insights.

This study assesses the important determining service attributes of FC satisfaction and their asymmetric relationships in community-based dementia care center as a case study, using IRPA and IAA approaches [17]. The objectives were: (i) to compare the performance of each service dimension with the impact on service satisfaction through IRPA, and (ii) to recognize the effect of each attribute on FCs’ experienced satisfaction by calculating and interpreting the impact asymmetry through IAA. The significance of this research is to determine the main service attributes of CBDC so that service managers can develop more quality care strategies and allocate resources effectively.

## Methods

### Study design and data collection

A pioneering study that examined family caregivers' perceptions of the quality of care provided by community-based dementia care centers. A structured questionnaire is used to measure caregivers' satisfaction with service quality to assess the gap between perceived and expected quality. An onsite survey was conducted with them for 4 months (September–December 2019), at eight community-based dementia care centers in Taiwan. These centers were selected because they were under the guidance of the affiliated hospital. Potential participants were approached by a well-trained nurse, who outlined the purpose of the study, and invited them to participate in the survey. The participants were provided with a self-administered questionnaire after they gave their consent.

We recruited caregivers of patients with dementia at eight dementia centers in one city using the convenience sampling method. The sample size of each center ranges from 15 to 20, a total of about 155 samples. Inclusion criteria is participants in this study were FCs of patients with dementia, who were receiving LTC. Exclusion criteria were (1) unwillingness to participate after being fully informed, and (2) less than 1 month of use. Since variables of expected and perceived quality satisfaction were used as independent and dependent variables in the analysis, those who have incomplete data (more than half of the missing value are in each quality dimension) were then be excluded. In total, 125 questionnaires were distributed, and after eliminating questionnaires with incomplete data, 95 of these were included in data analysis.

Regarding the sample size for factor analysis, there are two major recommendations. These include samples with less than 100 samples should have a factor loading of no less than 0.50 [18] and the subject-to-variable ratio of at least 10 cases for each item in the instrument being used [19]. The effective sample size in this study is 95, which is the subject-to-variable ratio of 23 for each item, and the factor loading is over 0.7. We analyzed the data of 95 samples with statistical power.

### Measures

The questionnaire was designed based on Service Quality (SERVQUAL) model; [20] this model is recommended as a good scale to use when measuring service quality [21]. Structure questionnaire was used to survey the FCs of dementia patients who were receiving LTC. It is divided into: (A) Demographic information—age, gender, education, marital status, and occupation; (B) Community care expectations and perceived performance was assessed using the expanded SERVQUAL Scale [20, 22]. The SERVQUAL scale evaluates five dimensions of community care service quality—tangibility, reliability, responsiveness, assurance, and empathy—using 20 items with a 5-point Likert response scale 1- strongly disagree and 5-strongly agree. The questionnaire demonstrated (i) adequate content validity indices (CVIs > 0.090) for all five dimensions as verified by 3 experts and (ii) adequate reliability through high internal consistency for all five dimensions (all Cronbach’s α > 0.90). The scale demonstrated adequate construct validity and acceptable reliability for this study.

### Analytical framework

All data collected were statistical analyzed using SPSS version 22.0 (IBM Corp, Armonk, NY, USA). Descriptive statistics, chi-square statistics, Pearson correlation, t-test, and one-way analysis of variance (ANOVA) were used to analyze the data. The analytical framework consists of three steps:

### Step 1: A penalty-reward contrast analysis (PRCA) [23]

A preliminary step in IRPA is the penalty-reward-contrast analysis (PRCA) which is a multiple regression analysis with dummy variables [23]. Due to the nonlinear effect, dummy variables were adopted to analyze the nonlinear relationship between the performance of quality attributes and customer satisfaction [24, 25]. The logistic regression was developed to describe the nonlinear relationship [26], to infer the odds ratio of customer satisfaction to customer dissatisfaction due to quality attribute performance, and to analyze the influence of quality attribute performance on customer satisfaction. Analyzing quality attributes by using quantified odds facilitates better understanding customer satisfaction.

For each quality attribute, two sets of dummy variables were created. In the first set, the lowest performance score was coded as “1” (if attribute = 1), and all other ratings were coded “0” (if attribute = 2, 3, 4, and 5). Conversely, in the second set, the highest performance ratings were coded as “1” (if attribute = 5), whereas all other ratings were coded 0 (if attribute = 1, 2, 3, and 4). These two dummy sets were then regressed on CS, which resulted in two unstandardized coefficients (penalty and reward indices) for each attribute [27]. These reward indices (RI) and penalty indices (PI) identify whether a service quality attribute plays a significant role in customer satisfaction or dissatisfaction respectively.

### Step 2: An impact range-performance analysis (IRPA) [17]

The next step is to calculate the range of each attribute's impact on customer satisfaction by summing up the absolute values of the PIs and RIs. The sum of the absolute value of the penalty index (PI) and the reward index (RI) for each service quality attribute was used to evaluate the attribute's range of impact on customer satisfaction (RICS). Then, PI, RI and RICS were used to calculate scores of impact-asymmetry (IA) that quantified the extent to which an attribute had a satisfaction-generating potential (SGP) compared to its dissatisfaction-generating potential (DGP). According to Mikulic and Prebežac [11], the following equations were used:

1. SGPi = Ri / RICSi … (1)
2. DGPi =|Pi| / RICSi … (2)
3. IAi index = SGPi – DGPi … (3)

in which:

1. ri = reward index for attribute i;
2. pi = penalty index for attribute i;
3. RICSi =|Pi|+ Ri = range of impact on overall customer satisfaction; and
4. SGPi + DGPi = 1.

A two-dimensional grid, divided into four quadrants, was constructed with the scores of an attribute’s range of impact on customer service (RICS) on the X-axis and mean values of attribute-performance scores (APS) on the Y-axis. The improvement-priority increases with larger RICS and lower APS [11].

Step 3: An impact-asymmetry analysis (IAA) [11, 28]

IAA is used to explore the key determinants of customer satisfaction/dissatisfaction among dementia care service quality attributes. By using grand mean values of IA (y-axis) and RICS (x-axis), the relative positioning of each attribute with the gridlines was provided IAA. Since IA is the arithmetic difference between SGP and DGP, IA can be used as a standard for classifying various levels of service attributes. For example, if an attribute had a positive value of IA, the attribute can be classified as a satisfier or delighter. In contrast, if the IA value of the attribute was negative, it is classified as a dissatisfied or frustrator. However, if the value of the attribute was close to 0, it can be classified as a hybrid because the attribute has little effect on customer satisfaction and dissatisfaction. The X-axis was divided into five parts, based on the degree of impact asymmetry on overall satisfaction: (i) “delighters” (Impact Asymmetry Index [IAI] > 0.4), (ii) “satisfiers” (0.4 ≥ IAI > 0.1). (iii) “hybrids” (0.1 ≥ IAI ≥ –0.1), (iv) “dissatisfiers” (–0.1 > IAI ≥ –0.4), and (v) “frustrators” (IAI < –0.4). In addition to using IA scores, RICS values are set as per the distribution of attributes: (i) “high-impact attributes” (RICS_tangibles_ > 0.57, RICS_reliability_ > 0.73, RICS_responsiveness_ > 0.58, RICS_assurance_ > 0.41, RICS_empathy_ > 0.71); (ii) “medium-impact attributes” (0.45 < RICS_tangibles_ ≦ 0.57, 0.49 < RICS_reliability_ ≦ 0.73, 0.38 < RICS_responsiveness_ ≦ 0.58, 0.26 < RICS_assurance_ ≦ 0.41, 0.33 < RICS_empathy_ ≦ 0.71); and (iii) “low-impact attributes” (RICS_tangibles_ ≦ 0.45, RICS_reliability_ ≦ 0.49, RICS_responsiveness_ ≦ 0.38, RICS_assurance_ ≦ 0.26;,RICS_empathy_ ≦ 0.33) [12, 17].

## Results

### Participant Characteristics

Table 1 presents a description of the study sample demographics. As shown in Table 1, 67% of the participants were female and more than 60% were under 59 years old. More than 72.7% had attained upper secondary level education or higher, with 27.4% having attained tertiary level education or higher. Approximately 37.9 of the respondents have full-time work, 24.2% were retired. Most FCs were related to the patients of dementia as daughters (30.5%), followed by spouses (24.2%), sons (21.1%), daughters-in-law (15.8%), and other relatives (8.4%).

**Table 1:** Demographic characteristics of respondents

	Frequency	Percent
**Gender**		
male	31	32.6
female	64	67.4
**Age**		
age < 50	26	27.4
age 50–59	31	32.6
age 60–69	22	23.2
age > = 70	16	16.8
**Education**		
no formal education	6	6.3
primary school	12	12.6
secondary school	29	30.5
diploma	22	23.2
higher education	26	27.4
**Occupation**		
full-time work	36	37.9
part-time work	8	8.4
retirement	23	24.2
others	28	29.5
**Relationship with the case**		
spouse	23	24.2
daughter	29	30.5
son	20	21.1
daughter in law	15	15.8
others	8	8.4

### Result of impact of range of performance analysis (IRPA)

The IRPA uses stated performance as well as implied importance to identify the impact of service attributes on satisfaction. As stated in the description of IRPA, both RI and PI were calculated by using dummy variables and regression analysis (see steps 1 and 2 in analytical framework). In Table 2, RICS indicates the sum of RI and PI for each service attribute. PI, RI, and RICS are then used to calculate the SGP, DGP, and IA of each attribute.

**Table 2:** Attribute impact-range and asymmetry of services

		**RI**	**PI**	**RICS**	**SGP**	**DGP**	**IA index**	**APS**	**Classification**	**Impact**
Tangibles										
1	Barrier-free environment	0.255	-0.375	0.635	0.402	0.598	-0.197	4.32	Dissatisfier	High
2	Neat and tidy appearance	0.251	-0.129	0.381	0.659	0.341	0.318	4.56	Satisfier	Low
3	Fire and Safety compliance	0.205	0.375	0.580	0.353	0.647	-0.293	4.52	Dissatisfier	High
4	Comfortable environment	0.144	-0.302	0.444	0.324	0.676	-0.351	4.63	Dissatisfier	Low
Reliability										
5	Appropriate services	0.187	-0.677	0.867	0.216	0.784	-0.569	4.59	Frustrator	High
6	Concern services	0.213	0.494	0.707	0.301	0.699	-0.397	4.57	Dissatisfier	Median
7	Service attitude	0.089	-0.324	0.409	0.218	0.782	-0.565	4.68	Frustrator	Low
8	Professional care	0.057	0.336	0.393	0.145	0.855	-0.710	4.63	Frustrator	Low
Responsiveness										
9	Care-giving processes	0.204	0.400	0.604	0.338	0.662	-0.325	4.53	Dissatisfier	High
10	Timely service	0.191	0.483	0.674	0.283	0.717	-0.433	4.6	Frustrator	High
11	Consultation services	0.239	0.161	0.400	0.598	0.403	0.195	4.51	Satisfier	Median
12	Care skills	0.207	0.066	0.273	0.758	0.242	0.516	4.56	Delighter	Low
Assurance										
13	Reliable services	0.149	-0.304	0.449	0.332	0.668	-0.336	4.66	Dissatisfier	High
14	Sense of security	0.182	0.000	0.182	1.000	0.000	1.000	4.73	Delighter	Low
15	Good manners	0.191	-0.036	0.231	0.827	0.173	0.654	4.69	Delighter	Low
16	Professional knowledge	0.174	0.314	0.488	0.357	0.643	-0.287	4.66	Dissatisfier	High
Empathy										
17	Special needs care	0.093	0.104	0.197	0.472	0.528	-0.056	4.46	Hybrid	Low
18	Patient-centered care	0.196	-0.042	0.236	0.831	0.169	0.661	4.49	Delighter	Low
19	Patient privacy	0.127	-0.315	0.447	0.284	0.716	-0.432	4.51	Frustrator	Median
20	Demand coordination	0.154	-0.806	0.964	0.160	0.840	-0.680	4.52	Frustrator	High

*RI* Reward index, *PI* Penalty index, *RICS* Range of impact on customer satisfaction (|PI|+ RI), *SGP* Satisfaction-generating potential (RI/RICSi), *DGP* Dissatisfaction generating potential (|PI|/RICSi), *IA* Impact-asymmetry (SGPi-DGPi), *APS* Attribute performance score

**Fig. 1:**
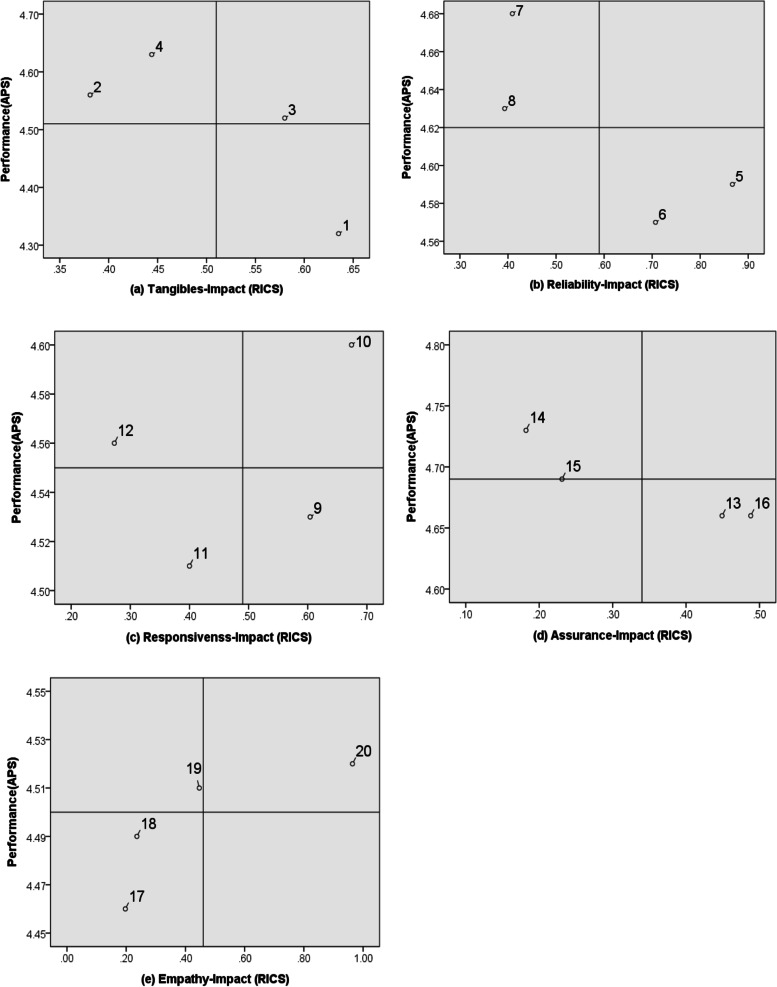
Impact-range1-performance analysis (IRPA) grid. The most important attributes are 1, 5, 6, 9, 13, and 16 (with low APS and high RICS); the attributes of medium importance are 3, 10, and 20 (with high RICS and high APS); and the attributes of low importance are 2, 4, 7, 8, 12, 14, 15, and 19 (with low RICS and high APS)

The further step of IRPA uses the performance score of each attribute and its RICS to position them in a grid (Fig. 1). For example, the four quadrants within the tangibles were distinguished using the grand mean values of the APS (4.51) and the RICS (0.51) scores. Figure 1a shows that attribute 1 (barrier-free environment) should be reviewed carefully because it was significantly lower than the grand mean of the APS score; it has a low APS score (4.32) and the highest RICS (0.635) score (see Table 2). Overall, Fig. 1 reveals that higher attention should be directed toward attributes 1 (barrier-free environment), 5 (appropriate services), 6 (concern services), 9 (care-giving processes), 13 (reliable services), and 16 (professional knowledge), because their performances (APS scores) are below average, but they have above average RICS values. On the other hand, attributes 3 (fire and safety compliance), 10 (timely service), and 20 (demand coordination) should be given medium priority because their RICS values and performance (APS scores) are above average. Lastly, attributes 2 (neat and tidy appearance), 4 (comfortable environment), 7 (service attitude), 8 (professional care), 12 (care skills), 14 (sense of security), 15 (good manners), and 19 (patient privacy) should be of lowest priority because their RICS values are below average, while their performance (APS score) is above average.

### Result of impact-asymmetry analysis (IAA)

To compute the potential asymmetry effects of attributes on customer satisfaction, a satisfaction-generating potential (SGP) and dissatisfaction-generating potential (DGP) were arithmetically derived for each attribute (see Table 2). Additionally, the category of attributes (satisfiers, dissatisfiers, hybrids, frustrators, and delighters) were identified by computing impact asymmetry (IA) scores. Moreover, another two-dimensional grid was constructed, with RICS values (X-axis) and IA scores (Y-axis), where the iso-impact line was drawn at IA = 0.

**Fig. 2:**
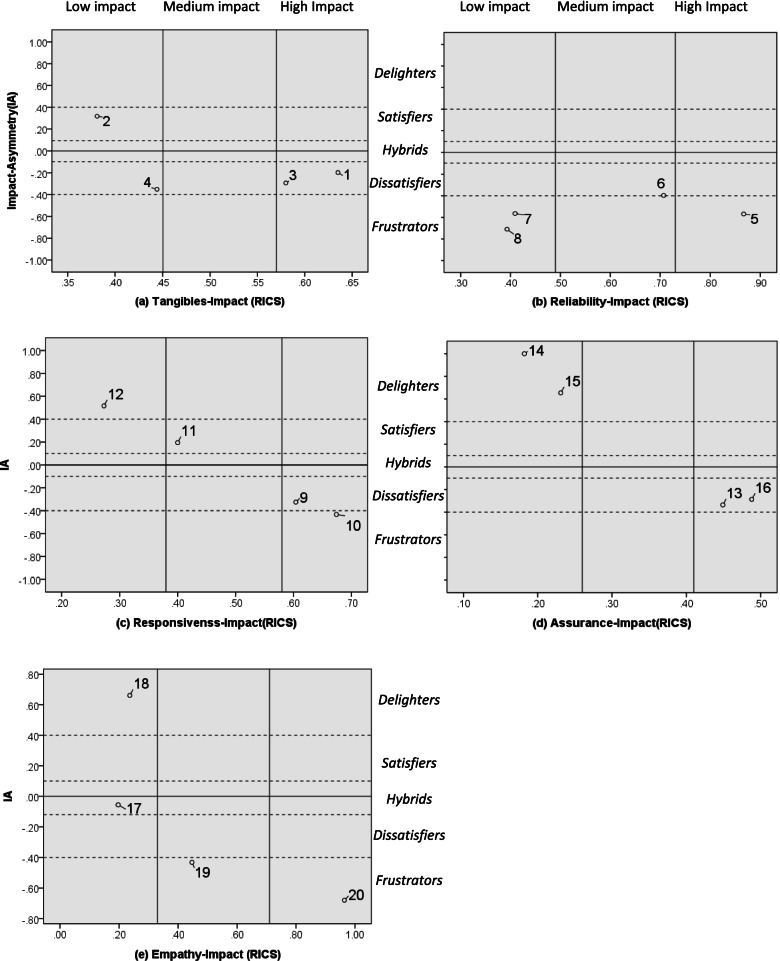
Impact-asymmetry analysis (IAA) grid. Attributes were categorized as delighters (12,14,15,18), satisfiers (2,11), hybrid (17), dissatisfiers (1,3,4,6,9,13,16), and frustrators (5,7,8,10,19,20) based on three levels of impact scores (high, medium, and low)

Figure 2(a-e) presents each factors’ IAA results. Among the tangible attributes (Fig. 2a), attributes 1 (barrier-free environment), followed by 3 (fire and safety compliance)—both categorized as dissatisfiers due to negative IA—had the greatest impact on RICS values. Furthermore, attribute 4 (comfortable environment) was identified as a dissatisfier due to low impact on customer satisfaction (RICS = 0.444), whereas attribute 2 (neat and tidy appearance) was classified as a delighter with minimal impact on CS (RICS = 0.381).

Among reliability attributes (Fig. 2b), attribute 5 (appropriate services) was categorized as a high impact frustrator (RICS = 0.867), attributes 7 (service attitude) and 8 (professional care) were low impact frustrators (RICS [7] = 0.409; RICS [8] = 0.393), and attribute 6 (concern services) was median impact dissatisfier (RICS = 0.707).

Regarding responsiveness attribute (Fig. 2c), attribute 9 (care-giving processes) was identified as a high impact dissatisfier (RICS = 0.604), whereas attribute 10 (timely service) was classified as a high impact frustrator (RICS = 0.674). Additionally, attributes 11 (consultation services) and 12 (care skills) were categorized as moderate impact satisfier and minimal impact delighter (RICS [11] = 0.400, RICS [12] = 0.273) for CS, respectively.

Among assurance attributes (Fig. 2d), attributes 13 (reliable services) and 16 (professional knowledge) were categorized as high impact dissatisfiers (RICS [13] = 0.449; RICS [16] = 0.488). However, attributes 14 (sense of security) and 15 (good manners) were recognized as high impact delighters, with low RICS impact (RICS [14] = 0.182; RICS [15] = 0.232).

Lastly, among empathy attributes (Fig. 2e), attribute18 (patient-centered care) was classified as a low impact delighter (= 0.197), whereas attribute 17 (special needs care) was identified as a low impact hybrid. Notably, attributes 19 (patient privacy) and 20 (demand coordination) were categorized as moderate (RICS [19] = 0.447) and high (RICS [20] = 0.964) impact frustrators for CS, respectively.

In summary, we obtained four delighters, two satisfiers, one hybrid, seven dissatisfiers, and six frustrators. Attributes have “medium impact “ (6,9) and “high impact” (1,3,5,9,10,13,16,20) on satisfaction and can easily generate dissatisfaction if the performance of this attribute is poor.

## Discussion

### Summary of research findings

CBDC does a great job of satisfying customers by offering multiple service attributes—the mean value of each service quality attribute performance score was around 4.6 on the 5-point Likert scale. This study examines strategies to improve community-based dementia care service quality using the IRPA and IAA approaches. The results reveal several relationships between CBDCS quality attributes and customer (family caregivers) satisfaction. According to the IRPA approach, the attribute with the strongest influence on caregivers' satisfaction is “Demand coordination” (RICS = 0.964), followed by “Appropriate services” (RICS = 0.867) and “Concern service” (RICS = 0.707). These attributes are related to empathy and reliability.

The IAA results indicate that attributes with a high impact on customer satisfaction include various frustrators and dissatisfied. This finding indicates that high performance affects customer satisfaction positively, whereas low performance affects customer satisfaction negatively. For example, the most and second influential attributes “Demand coordination” and " Appropriate service" are frustrators; thus, these attributes have a high impact on customer dissatisfaction when its performance is low. Dissatisfiers are attributes that negatively affect overall satisfaction when performance is low. As a dissatisfier, the attribute “Concern service” suggests that poor personalized service has a greater impact on dissatisfaction than its impact on satisfaction. Research into CBDCS quality evaluation has not previously revealed this finding. In light of this result, CBDC service should meet customer needs and fulfill customer preferences in order to achieve customer satisfaction.

### Theoretical implications

The applicability and practicability of this analytical framework is demonstrated in a case study of family caregivers’ satisfaction with community-based dementia care services. To avoid potentially misleading conclusions based on attribute-importance data, this study adopted the three-step analytical model to assess the extent to which an attribute contributes to the customer's judgment of the performance of the service [11, 12, 17]. The IRPA and IAA results indicated several priorities for the Taiwanese long-term care system to improve the service-related attributes of the dementia care service. It is suggested that this method can also be applied to other LTC service systems to facilitate service managers to make decisions about the improvement priority of service attributes.

To the best of our knowledge, this is the first study to classify the attributes that influence FCs satisfaction on community-based dementia care service as “delighters”, “satisfiers”, “hybrid”, “dissatisfiers”, and “frustrators”. With regard to community-based dementia care service evaluation in Taiwan, the IAA results show that the most influential attributes are those classified as “dissatisfiers” and “frustrators”. These attributes have been shown to contribute to customer dissatisfaction when their performance is low [29], and these dissatisfied customers are more likely to disseminate negative publicity [30]. Reducing unsatisfactory service attributes may effectively improve customers' overall satisfaction. Practitioners should be able to better determine the priority of resource allocation and formulate effective strategies.

### Practical implications

IRPA and IAA results indicated several priorities for improving service-related attributes of the Taiwanese long-term care industry. The study suggests that service managers should pay more attention to attributes with relatively high RICS and IA scores. For instance, barrier-free environment (1), fire and safety compliance (3), appropriate service (5), care-giving process (9), timely service (10), reliable services (13), professional knowledge (16), and demand coordination (20) should be carefully monitored since they had relatively high RICS and low IA. In other words, if service managers failed to provide good service quality on the above-mentioned attributes, customers were very upset with the overall service.

This study takes into account the asymmetric impact of attributes on customer satisfaction and provides detailed suggestions for enhancing FC satisfaction as well as eliminating FC dissatisfaction for service managers. The findings indicated that dissatisfiers—barrier-free environment (1), fire and safety compliance (3), care-giving process (9), reliable services (13), and professional knowledge (16)—emerged as most influential attributes, on the service quality survey, which contributed to caregiver dissatisfaction with the dementia care services. Service strategies should be applied to help reduce those dissatisfaction. Based on this finding, there is an urgent need to improve the tangibles services because barrier-free environment and safety compliance are of high importance in LTC facilities [31, 32]. Additionally, service managers should promote training programs for service assistant from dementia care centers, to develop prompt care-giving process for patient via coordination between supply and demand. The findings provide practical guidelines for identifying the impact of service attributes on dissatisfaction.

Our study further revealed several significant asymmetries in the relationship between IA and RICS. The results indicated particularly strong asymmetries with attributes 20 (demand coordination), 5 (appropriate service), and 10 (timely service). These three attributes have a much strong potential to cause dissatisfaction than satisfaction, so they were classified as frustrators. As the result that emerged from our study, what the family caregivers needed were the provision of caring and individualized attention for their loved one, however, the problem of service quality is mostly related to service management which do not focus on understanding and meeting customers’ needs and demands, this is in line with findings from previous studies [33, 34]. Since family caregivers of dementia are very concerned with demand coordination in dementia care service, service managers should communicate regularly with the customers, and quality coordination with caregivers in particular since they are the key person in deciding whether to continue using the service. A possible implementation strategy is to improve the actively to involve all caregivers and case assistants in the co-creating care provision process to assure service appropriateness for care planning, to enhance the customer satisfaction with service quality. This could be costly, but it would encourage continued service use by satisfying family caregivers. This involvement is especially important among countries with a high aging care requirement, such as Taiwan.

### Limitations

This study has several limitations. First, this study included participants from only selected community-based dementia care centers in a city of Taiwan. Thus, future studies should be conducted in other community-based dementia care centers to improve the generalizability of the findings, and to compare the similarities or differences between findings from different centers. Secondly, the cut-off criteria for classifying different attributes may vary based on the literature review. However, the current criteria were adopted from popular empirical research approaches. Lastly, this study examined informal caregivers’ perspectives. Future studies should examine and compare the perspectives of both formal and informal caregivers. This approach will enable researchers to develop a dynamic model to test the service quality of LTC 2.0.

## Conclusion

This study identifies particular attributes of community-based dementia care services of the existing LTC 2.0 system that require improvement in design and delivery by practitioners. The findings indicate a need for an increase in managers’ awareness of customer dissatisfaction; resource managers should mitigate this issue by strengthening the identified attributes. For this purpose, resource managers and family caregivers must work in close collaboration. Additionally, providing care, which is more tailored to the patients’ care needs and caregivers’ demand, may contribute to improved satisfaction. These combined efforts may help take another step forward in making LTC a more friendly system for older adults in Taiwan.

## Data Availability

The anonymized, non-identifiable data are available upon reasonable request. These survey data were collected under the CCH grant (CMC, principal investigator) for purposes of improving long-term care service quality for patient with dementia and reducing the burden of family caregivers. Requests should be directed to the corresponding author. Reuse is permitted for scientific purposes and quality initiatives.

## Abbreviations

APS: Attribute performance score
CBDC: Community-based dementia care
DGP: Dissatisfaction generating potential
FC: Family caregiver
IA: Impact-asymmetry
IAA: Impact Asymmetry Analysis
IRPA: Impact Range Performance Analysis
LTC: Long-Term Care
PI: Penalty index
RI: Reward index
RICS: Range of impact on customer satisfaction
SERVQUAL: Service Quality
SGP: Satisfaction-generating potential
