# Correction to: Evaluating caregivers’ service quality perceptions: impact-range performance and asymmetry analyses

**DOI:** 10.1186/s12913-022-07684-1

**Published:** 2022-03-02

**Authors:** Wen-Fu Wang, Chun-Min Chen, Kai-Ming Jhang, Yung-Yu Su

**Affiliations:** 1 Department of Neurology, Changhua Christian Hospital, Changhua, Taiwan; 2 Department of Recreation and Holistic Wellness, Ming Dao University, Changhua, Taiwan; 3 Big Data Center, Changhua Christian Hospital, Changhua, Taiwan; 4 Department of Long-Term Care, National Quemoy University, No. 1, University Rd., Jinning Township, Kinmen County 892, Kinmen, Taiwan, Republic of China

**Correction to: BMC Health Serv Res 22, 183 (2022)**

**https://doi.org/10.1186/s12913-022-07594-2**

Following publication of the original article [1], the authors identified an error in Fig. 2. The updated Fig. 2 provides a level illustration of Fig. 2. Several corrections have been made, including text above figure (Low impact, Medium impact, and High impact), and text in the center (Delighters, Satisfiers, Hybrids, Dissatisfiers, and Frustrators). The correct figure is given below.

**Fig. 2:**
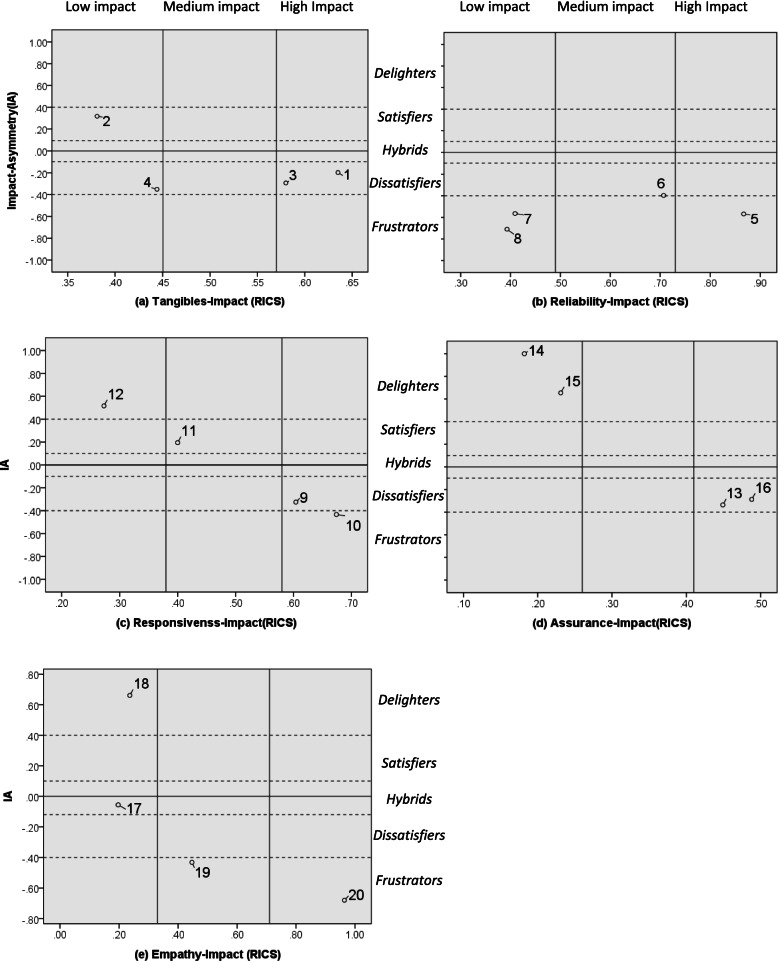
Impact-asymmetry analysis (IAA) grid. Attributes were categorized as delighters (12,14,15,18), satisfiers (2,11), hybrid (17), dissatisfiers (1,3,4,6,9,13,16), and frustrators (5,7,8,10,19,20) based on three levels of impact scores (high, medium, and low)

The original article has been corrected.
